# Chloroplast genome of tropical and sub-tropical fruit tree *Clausena lansium* (Rutaceae)

**DOI:** 10.1080/23802359.2018.1467217

**Published:** 2018-04-26

**Authors:** Ying-Feng Niu, Shu-Bang Ni, Zi-Yan Liu, Cheng Zheng, Chang-Li Mao, Chao Shi, Jin Liu

**Affiliations:** a Yunnan Institute of Tropical Crops, Xishuangbanna, PR China;; b Kunming Institute of Botany, Chinese Academy of Sciences, Kunming, PR China

**Keywords:** *Clausena lansium*, chloroplast genome, Rutaceae

## Abstract

The *Clausena lansium,* originally native to the southern part of China and has a long history of cultivation, is a tree member of the family Rutaceae. Chloroplast genome sequences play a significant role in the development of molecular markers in plant phylogenetic and population genetic studies. In this study, we report the complete chloroplast genome sequence of *C. lansium* for the first time (accession number of MH047377). The chloroplast genome is 159,284 bp long and includes 113 genes. It’s LSC, SSC, and IR regions are 88,634, 18,896, and 25,877 bp long, respectively. Phylogenetic tree analysis exhibited that *C. lansium* was clustered with other Rutaceae species with high bootstrap values.

Wampee (*Clausena* Burm. F.) is a tropical and subtropical, very remote citroid fruit tree belonging to subtribe Clauseninae, tribe *Clauceneae* of the family Rutaceae (Swingle and Reece 1967). Wampee has more than 30 species, 11 of which are native to China (Yu 1982). Among them, only *Clausena lansium* (Lour.) Skeels and *C. indica* (Dalz.) Oliv. are edible and commercially cultivated. Chinese wampee (*C. lansium*) was originally native to the southern part of China and has a long history of cultivation. Presently, its large commercial cultivation areas are the Guangdong, Guangxi and Fujian Provinces, and ‘Chicken Heart’ sweet wampee is the most famous cultivar. Wampee is a remote relative of *Citrus*, and sexual incompatibility exists between them (Guo and Deng 1999), but the phylogenetic relationship between them is unclear.

In this study, we report the complete chloroplast genome of *C. lansium*, the second complete plastome sequence from the genus *Clausena*. DNA material was isolated from mature leaves of a *C. lansium* plant cultivated in the plant garden of Yunnan Institute of Tropical Crops (YITC), Jinghong, China by using DNeasy Plant Mini Kit (QIAGEN, Germany). A specimen of this tree and the isolated DNA were stored in YITC. About 10 μg isolated DNA was sent to BGI, Shenzhen for library construction and genome sequencing on the Illumina Hiseq 2000 Platform. After genome sequencing, a total of 4.5 Gbp reads in fastq format were obtained and subjected to chloroplast genome assembly. The complete chloroplast genome was annotated with Dual Organelle GenoMe Annotator (DOGMA; Wyman et al. 2004) and submitted to the Genbank under the accession number of MH047377. Our assembly of the *C. lansium* resulted in a final sequence of 159,284 bp in length with no gap. The overall A–T content of the chloroplast genome was 61.1%. This chloroplast genome included a typical quadripartite structure with the Large Single Copy (LSC), Small Single Copy (SSC), and Inverted Repeat (IR) regions of 88,634, 18,896, and 25,877 bp long, respectively. Genome annotation showed 113 full length genes including 79 protein-coding genes, 30 tRNA genes, and 4 rRNA genes. The genome organization, gene content, and gene relative positions were almost identical to the previously reported Rutaceae chloroplast genomes (Liu and Shi 2017).

To validate the phylogenetic relationships of *C. lansium* in the Rutaceae, we constructed a maximum likelihood tree using 20 Rutaceae taxa. Phylogenetic analysis was performed on a data set that included 79 protein-coding genes and 4 rRNA genes from the 20 selected taxa using RAxML v. 7.7.1 (Stamatakis et al. 2008). The 83 gene sequences (82,566 bp in length) were aligned with the MAFFT (Katoh and Standley 2013). The resulting tree shows that *C. lansium* forms a clade with the species of *C. excavata*, and sister to *Murraya koenigii* with a 100% bootstrap value (Figure 1).

**Figure 1.**
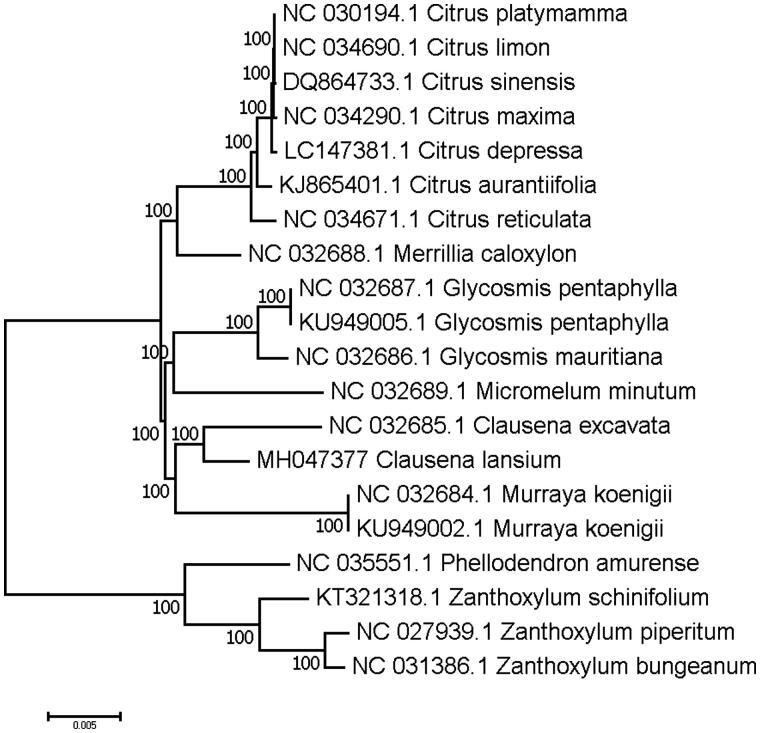
Maximum-likelihood (ML) phylogenetic tree of *C. lansium* in Rutaceae. Number above each node indicates the ML bootstrap support values.
